# “That was intense!” Spirituality during childbirth: a mixed-method comparative study of mothers’ and fathers’ experiences in a public hospital

**DOI:** 10.1186/s12884-016-1072-z

**Published:** 2016-09-30

**Authors:** Marie-Noëlle Bélanger-Lévesque, Marc Dumas, Simon Blouin, Jean-Charles Pasquier

**Affiliations:** 1 Centre d’études du religieux contemporain, Université de Sherbrooke, Sherbrooke, Québec Canada; 2 Departement of Obstetrics and Gynecology, Faculty of Medicine and Health Sciences, Université deSherbrooke, 3001, 12ème avenue Nord, Sherbrooke, Québec JIK 2R1 Canada; 3 Centre de recherche du Centre hospitalier universitaire de Sherbrooke, CRCHUS, Sherbrooke, Québec Canada

**Keywords:** Spirituality, Birth, Mothers, Fathers, Mixed-methods study

## Abstract

**Background:**

While spirituality is well described in end-of-life care literature, research on its place in the delivery room remains largely limited to mother-oriented qualitative studies focusing on life-threatening situations (e.g., high-risk pregnancies). Our aim was to compare mothers’ and fathers’ spirituality during childbirth.

**Methods:**

A mixed methods questionnaire was developed from our childbirth-related spirituality categorization and distributed to all parents of newborns, 12–24 h postpartum, over 45 consecutive days. Paired-sample t-tests and qualitative thematic analysis were used to compare mothers and fathers. Multiple linear regressions identified factors associated with their respective global scores (vaginal and cesarean deliveries separately).

**Results:**

The global scores for mothers (38.6/50) and fathers (37.2/50) were similarly high (*N* = 197; *p* = 0.001). Highest-ranked (“respect”, “moral responsibility”, “beauty of life”, “gratitude”) and lowest-ranked spiritual themes (“prayer”, “greater than self”) were in agreement. Fathers scored higher on “fragility of life” (*p* = 0.006) and mothers on “self-accomplishment” (p‹0.001), “letting go” (p‹0.001), and “meaningfulness” (*p* = 0.003). “Admission of baby in neonatal unit” was associated with higher global score for both mothers and fathers. Other factors also increased fathers’ (witnessing a severe tear) and mothers’ scores (birthplace outside Canada; for vaginal deliveries, religious belonging and longer pushing stage).

**Conclusion:**

These first quantitative data on the prevalence of spirituality during childbirth highlight a high score for both parents, among a non-selected public hospital population. Spirituality emerges not only from unordinary situations but from any childbirth as an “intensification of the human experience”. Significant differences for some spiritual themes indicate the need to consider the spirituality of both parents.

## Background

Childbirth is a multidimensional event and is regarded as one of life’s most important experiences. Nonetheless, and despite a growing body of literature establishing that all hospitalized patients potentially have spiritual needs [1], studies on spirituality in health care do not tend to focus on the beginning of life, but rather on end-of-life or life-threatening experiences, such as oncology and palliative care [2].

Establishing a consensual definition of spirituality, which is understood as distinct but not in contradiction to religion, has been a matter of debate in the literature. On the one hand, clarity and consensus on a definition could help to investigate and conceptualize the interdisciplinary roles and provision of spiritual care [2]. On the other hand, it has been said that we “should not impose a view or definition of spirituality, but should seek to elicit the thoughts, memories and experiences that give coherence to a person’s life” [3]. While no universally accepted definition can be given to this subjective notion, developed typologies are based on its multidimensionality and its concern “with the meaning of life, individual perceptions of faith, and an individual’s relationship with the Ultimate Source” [4].

The most recent literature underlines the importance to educate childbirth health professionals on spirituality [5, 6]. However, existing studies on spirituality surrounding childbirth focus on unordinary situations: infertility [7, 8], homebirths [9], high-risk pregnancies [10], delivery after miscarriage [11], extremely low weight infant [12], death of a twin [13], or postpartum depression [14]. This recurrent interest to study spirituality in more pathological contexts, notably as a coping mechanism, aligns with the end-of-life care literature and rare are the studies addressing the positive spiritual dimension of such life-creating events [15, 16]. Moreover, the current literature limited to mother-oriented qualitative studies in specific medical and/or cultural groups, crucial to comprehend this topic but insufficient to provide a complete picture of the topic impossible without quantitative data or studies with large non-selected populations. Fathers are also underrepresented in studies related to spiritualityy, [17], as they are now pivotal actors in the delivery room and should be considered as parents with their own unique experiences and needs, and not only as the mother’s partner [18, 19].

The aim of this interdisciplinary study was to compare the childbirth spiritual experiences of mothers and fathers, and to correlate a spirituality score with different sociodemographic and birth medical data.

## Methods

### Study design and population

Quantitative approach to spirituality is difficult and we developed an original design to encompass more widely and completely this variable in its complexity by associating quantitative and qualitative data. Our choice of a simultaneous mixed methods design with a deductive theoretical drive, as described by Morse et al. [20] is hencebased more heavily on the acquisition of quantitative data while been supplemented by a qualitative description.

After approval by our institution’s Research Ethics Board (09–148), an exhaustive survey was conducted between May 15th and June 30th, 2012, at the Centre hospitalier universitaire de Sherbrooke (CHUS; Sherbrooke, Canada). Some characteristics of this public hospital are: 1) sole maternity of the region (level III/subspecialty maternal care, with 2851 births in 2014); 2) deliveries equally performed by general practitioners and obstetricians; 3) full-time attending nurse during labor; 4) delivery rooms where women remain until the end of their stay; 5) well implanted skin-to-skin practice (in development for cesarean); 6) general practice based on the Society of Obstetrics and Gynecologists of Canada guidelines. Relevant Quebec provincial characteristics to situate this study are: 1) universal public health care coverage; and 2) largely decreased religious practice (traditionally Roman Catholic) but diversification of spiritual beliefs [21].

Parents were recruited postpartum, at least 12 h after a vaginal delivery and 24 h after a cesarean delivery. Here “mother” refers to the woman giving birth and “father” refers to the father of the child or the mother’s life partner (male or female) who was present at the birth. Parents had to be able to read and speak French or English. Cases of major infant malformations or death were excluded.

### Ethics, consent and permissions

Participating parents signed an informed and consent form, which informed them of the voluntary nature of their participation and of the study’s aims, procedures, and data confidentiality (anonymous codification). Authorization to consult the birth medical records was also requested. Surveys were returned in envelopes before the end of hospitalization.

### Data collection

Quantitative and qualitative components were conducted simultaneously through a self-administered survey given to each parent. To measure the “spirituality” dependent variable, we built on a previous study of our research program involving a categorization of birth-related spirituality based on the literature and multidisciplinary expert meetings and focus groups (parents, nurses, doctors, midwives, and theologians) [14]. The ten identified spiritual themes were: 1) relational aspects (respect, trust); 2) gratitude and hope; 3) control/letting go; 4) death/resolution; 5) belonging/greater than self; 6) capacity to appreciate beauty; 7) transcendence; 8) moral and ethical aspects; 9) self-awareness and self-accomplishment; and 10) meaningfulness. The quantitative core component of this study is the conversion of these themes into simple affirmations using the first-person pronoun, with a 5-point Likert scale generating a maximum score of 50. As for the qualitative component of the study, a comment section was placed under each statement. Versions of the survey in French and English were produced using the appropriate method [22, 23]: 1) translation and back-translation carried out separately by two translators; 2) evaluation by the research committee; and 3) pilot testing with parents of newborns (*n* = 25) to assess the clarity of the questions.

The following independent variables were measured: i) sociodemographic data via the survey and ii) birth medical data via the electronic form of the mother’s medical record. Most variables were categorical, and a few were continuous (i.e., length of different birth stages, parent’s age, parity).

### Analysis

The sample size (*N* = 200) was based on the satisfaction section of the survey –another theme explored in the survey but whose results were analyzed separately and previously published [24] – since no previous quantitative study was made previously to allow a predicted difference. Quantitative and qualitative components were analyzed separately and integrated for discussions. Statistical analyses of the quantitative data were performed using SPSS 18.0 software. Means and standard deviations were given for variables on a continuous scale and a percentage was given for categorical variables. Questionnaires with less than 8 out of 10 questions answered were not given a total score (maximum = 50); others were recalculated proportionally. Paired-sample t-tests were used for comparing mothers’ and fathers’ global spirituality and thematic scores. As for factors predicting mothers’ and fathers’ global spirituality scores, vaginal deliveries and cesareans were analyzed separately, since many medical variables were not shared. Simple linear regression analysis was first performed to identify significant variables (*p* < 0.10), which were then entered in different multiple linear regressions (forward stepwise method).

For the qualitative section, typology development was selected as an analytic integration [25], with the specific use of a six-phase thematic analysis method: 1) data familiarization; 2) generation of initial codes; 3) themes search; 4) themes review; 5) themes definition and naming; and 6) report [26]. In this article, French comments have been translated; all comments are available in their original language for consultation.

## Results

### Sample description

Among 353 eligible births during the period covered, 228 mothers (64.6 %) and 204 fathers (57.8 %) completed the questionnaire. For the purpose of this study, data were analyzed only when both parents participated, for a total of 197 couples. Comments sections were completed by 2 to 10 % of parents, depending on the questions. Table 1 presents pparental characteristics and Table 2 delivery characteristics.

**Table 1:** Parents characteristics at the time of delivery

Characteristics	Mothers	Fathers
Age, mean (SD)	28.7 (4.88)	31.1 (4.88)
Marital Status, % (n)		
Married	23.4 (46)	23.5 (46)
Common Law Spouse	72.6 (143)	73.5 (144)
Other	4.0 (8)	3.0 (6)
Education level, % (n)		
Primary	1.0 (2)	3.1 (6)
Secondary	17.3 (34)	22.4 (44)
Professional Studies diploma	16.3 (32)	27.1 (53)
College	26.0 (51)	15.8 (31)
University	39.3 (77)	31.6 (62)
Working status, ^a^ % (n)	85.8 (169)	95.4 (188)
Religious affiliation, % (n)	54.7 (105)	51.8 (100)
Born in Canada, % (n)	91.9 (181)	92.3 (179)

^a^Before pregnancy for mothers. Owing to missing data, the numbers for each variable might not add up to the same total

**Table 2:** Delivery and infant characteristics

	Prevalence of characteristic	
Characteristics	Number	Percent
Type of delivery		
Spontaneous vaginal delivery	142	72.1
Instrumental delivery	12	6.1
Breech vaginal delivery	1	0.5
Primary cesarean	27	13.7
Iterative cesarean	15	7.6
Gestational weeks < 37 weeks	13	6.6
Primiparity	75	38.9
Anesthesia		
None	22	11.5
General	3	1.6
Epidural	119	62.0
Rachidian	19	9.9
Pudendal block	17	8.9
Local	18	9.4
Episiotomy^a^	17	11.1
Tear^a^		
None	31	20.7
Periurethral	56	37.8
Vaginal	14	9.5
Perineal 1^st^ degree	16	10.8
Perineal 2^nd^ degree	43	29.1
Perineal 3^rd^ degree	9	6.1
Uterine exploration^a^	13	9.8
Blood loss, > 500 mL	42	22.5
Fetal monitoring anomalies	39	23.9
Birth weight < 2500 g	7	4.4
Neonatology admission	23	12.0
Apgar < 7		
At 1 min	19	10.1
At 5 min	3	1.6

Owing to missing data, the numbers for each variable might not add up to the same total

^a^Percentage calculated for vaginal delivery only

### Identical highest-ranked spiritual themes between mothers and fathers

**Fig. 1:**
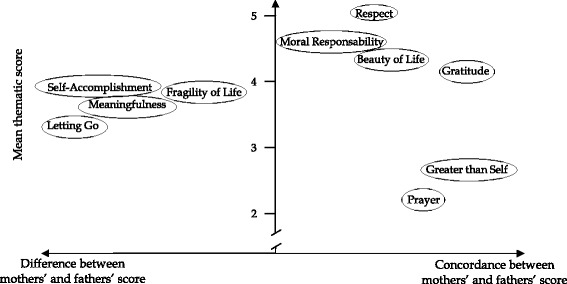
Mothers’ and fathers’ childbirth spiritual experience: concordance and mean thematic score

Mothers' and fathers' scores showed some concordance on some spiritual themes and differences for others (Fig. 1).

Quantitative data showed agreement between mothers and fathers for the four highest-ranked spiritual themes (Table 3). These themes are presented from highest to lowest-ranked, with a description of the comments made by parents.

**Table 3:** Mothers’ and fathers’ spirituality scores, mean difference, and significance by paired-sample *t*-test

Questions	Spirituality score, average (SD)				
	Mothers	Fathers	Mean difference	*t*	*P*
1. I felt respected and accepted as I am.	4.7 (0.57)	4.6 (0.57)	0.09	1.83	.068
2. I felt immensely grateful.	4.2 (0.79)	4.1 (.76)	0.08	1.26	.210
3. I was able to let go in the delivery room.	3.6 (1.01)	3.3 (0.98)	0.36	3.85	*.000*
4. I became aware of life’s fragility.	3.8 (1.06)	4.1 (0.91)	−0.24	−2.80	*.006*
5. I was guided by Something or Someone greater than me.	2.7 (1.26)	2.6 (1.26)	0.08	0.78	.435
6. I was able to appreciate life’s beauty.	4.5 (0.62)	4.4 (0.74)	0.10	1.62	.107
7. I prayed in the delivery room.	2.2 (1.38)	2.1 (1.33)	0.11	105	.297
8. I felt deeply responsible for this naked child.	4.6 (0.67)	4.4 (0.77)	0.16	2.16	*.032*
9. This birth allowed me to surpass myself, to fulfill myself.	4.2 (0.85)	3.8 (0.94)	0.38	4.69	*.000*
10. I was able to make sense of this childbirth experience.	4.1 (0.83)	3.8 (0.96)	0.25	3.02	*.003*
Total score (maximum 50 points)	38.6 (4.55)	37.2 (4.93)	1.30	3.40	*.001*

“Relational aspects” (Q1) Comments made for the highest-scored theme were largely very positive for both mothers and fathers. Most comments were related to staff acceptance (or not) of specific issues. For example, a lesbian co-mother wrote, “I was accepted as the mother’s partner and second mother by all the staff.”

“Responsibility for the child” (Q8) This measure of the “moral/ethical” spiritual theme was shared by parents as the second highest-ranked theme, with mothers scoring slightly but significantly higher (*p* = 0.032). This responsibility was considered obvious: “I am the mother” or “Our infant is a very precious being”. Some fathers emphasized their role as “protector”. A few fathers and one mother joked about the statement “I felt deeply responsible for this naked child” by stating that even if “clothed” they would have felt the responsibility. Among the small number disagreeing with the statement, one mother specified that her responsibility was delayed since her child was taken in charge in the neonatal unit and another said she felt “powerless”.

“Capacity to appreciate beauty” (Q6) Both parents considered childbirth as “magical” and “magnificent”, and “giving life” as “the most beautiful thing that can happen”. They were moved by their first contact with the baby (first cry, skin-to-skin) or by the beauty of nature. Many fathers specified that this appreciation of life’s beauty was not new to them as they had felt it before and few others stated not being able to appreciate beauty just yet.

“Gratitude and hope” (Q2) Half of the comments made were directed toward the staff. Most comments were positive with mothers saying they felt “understood”, but some indicated variations between health professionals. One woman expressed her gratitude toward all present at the birth. One father stated his gratitude toward the mother, specifying that her role was greater than his own supporting, but limited, role.

### Identical lowest-ranked spiritual themes between mothers and fathers

Themes related more directly to the connection with transcendence were the two lowest-ranked themes (Table 3).

“Belonging/greater than self” (Q5) Experience of guidance by “greater than oneself” was agreed or strongly agreed by 31.8 % of mothers and 27.3 % of fathers. It was the theme with the most comments and the most diverse opinions. Some fathers straightforwardly wrote down “God” and one mother talked about “Someone” being there. Some parents disagreed, clearly stating they were atheist or without a religious practice. Most comments designated this guide “greater than oneself”, which was either the mother’s mother, a member of the medical staff, the father, or one’s own baby. The designation of the same person sometimes generated opposite quantitative answers about transcendence:for example, if two mothers indicated their partner, one agreed with the statement, while the other totally disagreed. Similarly confusing, some mothers indicated not having been guided by something greater than self but rather by “instinct”, “hope” or an inside force: “It’s a force inside of me that guided me, a hidden force that I went to meet, that I called and that guided me, and that shined on everyone witnessing my birth.” Some fathers stated to rather rely on their own mental state to live “one thing at a time”. Some fathers joked with the double meaning of “*plus grand*”, which means both “greater” and “taller” in French, by stating that they were the “great/tall” one in the room. One father felt that this question “does not have its place in such a survey”.

“Transcendence/prayer” (Q7) Even as the lowest-ranked theme, 23.6 % of mothers and 19.6 % of fathers agreed or strongly agreed to having prayed in a public hospital setting and this was highly commented. Some parents said they prayed because of a dangerous medical situation (e.g., umbilical cord around neck). Others specified that they prayed specifically at the beginning of the delivery. Ambivalent results emerged: two fathers talked to their deceased father, one considered it praying whereas the other did not; two mothers indicated both “yes” and “no” in the comments section. Many parents clearly indicated not being “believers”. One father stated he would rather have “faith” in the medical team. Another father pointed out that “science had solved more problems than faith itself”. One father considered it an inappropriate question, and two others made a joke out of it.

### Significantly different middle-ranked spiritual themes between mothers and fathers

Middle-ranked spiritual themes emerged as the ones with the most important and statistically significant difference between mothers and fathers (Table 3).

“Fragility of life” (Q4) This is the only spiritual theme for which fathers had a higher score than mothers (*p* = 0.006). Fear was mostly expressed by fathers, but also by one mother, with some emphasis on the fragility of life. Nonetheless, some parents expressed the merits of modern medicine and trust toward the staff. Most comments by mothers, but also one father, mentioned the baby’s difficult medical condition (e.g., heart deceleration, umbilical cord around the neck) or the mother’s condition (hemorrhage, emergency cesarean). When no complications arose, mothers rather disagreed with the statement, emphasizing the “force of life” rather that its fragility: “Baby was very strong with no [cardiac] deceleration throughout the birth.”

“Self-awareness and self-accomplishment” (Q9) More mothers than fathers indicated that this experience allowed them to surpass/fulfill themselves (*p* < 0.001). Some mothers said that this was “one of life’s great moments” and others stated pride in themselves for “not giving up”, considering “the high level of pain” or the difficult medical decision to be taken (epidural anesthesia, cesarean). Nonetheless, few mothers felt overwhelmed. Fathers generally pointed out their ability to stay calm or emphasized the mother’s accomplishment, or their joint accomplishment as a couple. Some fathers described their experience as positive, but others saw that their accomplishment will come with the responsibility they will have to take toward this new baby. “Meaningfulness” (Q10) Mothers were significantly more able to make sense of childbirth than fathers (*p* = 0.003). Interestingly, fathers added comments three times more often as mothers, stating “pride toward the mother”, “I would not want to be elsewhere”, and the importance to “live up to challenges life gives you”. Fathers also talked about the beauty and importance of this moment that “gives meaning to life”. A few fathers seemed impatient with this last question, stating very clearly that they have no religious practice. Among mothers, one talked about her grief as this would be the last child of the family, while another indicated the importance of letting go and following her instincts.

“Control/letting go” (Q3) More mothers were also able to let go than fathers (*p* < 0.001). Among comments made by mothers, some talked about following the natural rhythm of birth and their ability to let go between contractions. In comparison, almost half of the comments by men stated that it was the father’s role not to let go and/or to be an active agent during the birth: “I invested myself in this birth, I did not let go.” A few fathers said they do not understand childbirth and relied on the professionals’ competence. Letting go was also associated with the decision to undergo a medical intervention by some parents, like this mother: “Thanks to the epidural anesthesia!”

### Global spirituality score and associated factors

Average global scores were 38.6 (4.55) and 37.2 (4.93) out of a total of 50 for mothers and fathers respectively, a significant difference (*p* = 0.001) with a moderate effect size (0.055). If analyzed separately for methodological reasons, the global spirituality scores between vaginal births and cesareans had no significant difference, for both mothers (*p* = 0.458) and fathers (*p* = 0.848). After initial simple linear regressions, three models emerged from the multiple linear regression analyses showing factors associated to the global score of: 1) mothers with vaginal delivery; 2) mothers with cesareans; and 3) fathers present at a vaginal delivery (Table 4). No significant model was obtained for fathers who witnessed a C-section.

**Table 4:** Factors significantly associated with the spirituality global score, by multiple linear regression

		Non standardized coefficients		Standardized coefficients			Model		
Group	Variable	B	SE B	*β*	t	*p*	F	*p*	Adjusted R^2^
Mothers - vaginal deliveries	Religious belonging	2.86	0.82	0.31	3.52	.001	4.86	.030	.217
	Length of second stage	0.02	0.01	0.24	2.76	.007			
	Neonatology admission	3.17	1.39	0.20	2.29	.024			
	Birth country	3.62	1.64	0.19	2.20	.030			
Mothers -cesarean deliveries	Birth country	4.86	2.37	0.31	2.05	.047	4.22	.047	.074
Fathers - vaginal deliveries	Neonatology admission	3.93	1.45	0.22	2.72	.007	4.61	.033	.065
	Perineal tear 3^rd^ degree	1.90	.89	0.18	2.15	.033			

For mothers with vaginal deliveries, we found that some sociodemographic variables correlate with a higher global spirituality score: i) belonging to a religious group (*p* = 0.001); and ii) being born outside Canada (*p* = 0.030). Specific difficult medical situations also increased the score: i) a longer pushing stage (*p* = 0.007) or 2) admission of the baby to the neonatal unit (*p* = 0.024). As such, the R^2^ explained by these four variables was 21.7 % for this group.

For mothers with cesareans, the last model also showed that mothers born outside Canada had a significantly higher score (*p* = 0.047), the R^2^ explained by this variable was 7.4 %. It should be indicated that, beside Canada, mothers’ countries of birth were distributed equally between the developed and the emerging world, and France was the only country mentioned more than once (*N* = 3). For all mothers, answers to questions 5 (greater than self) and 7 (prayers) played an important role in explaining significant differences among scores.

For fathers present at a vaginal delivery, the last model showed a higher global spirituality score when: i) a severe perineal tear occurred (*p* = 0.033), with higher scores for question 3 (letting go); and ii) the baby was admitted to the neonatal unit (*p* = 0.007), with higher scores for question 5 (greater than self). As such, the R^2^ explained was 6.5 %.

## Discussion

These first quantitative data from an exhaustive survey of the spiritual dimension of childbirth have shown a high mean score for both parents. When analyzed more closely, mothers’ and fathers’ scores were in agreement for the highest-ranked spiritual themes (“relational aspects”, “moral responsibility”, “capacity to appreciate beauty”, “gratitude”) and for the lowest-ranked themes (“prayer”, “greater than self”). Fathers scored higher for “fragility of life”, while mothers for “self-accomplishment”, “meaningfulness”, and “letting go”.

### Difficult childbirth and spirituality

Spirituality was shown to act in difficult situations which is consistent with its role as a coping mechanism established in the introduction [5]. As such, comments on the “fragility of life” reflect this connection to difficult medical situations for the baby (irregular heartbeat, cord around the neck) or for the mother (emergency cesarean, hemorrhage). Also, the global spirituality score was significantly higher when fathers witnessed a severe perineal tear or when mothers experienced a longer pushing stage. Moreover, we observed that a higher spirituality score could also result from the addition of stressful variables such as experiencing a C-section for mothers not born in Canada. Neonatal unit admission was the only significant variable common to both parents, confirming previous discussions in the literature. Catlin et al. research indicated that NICU (neonatal intensive-care unit) staff have stated that spirituality and religiosity have a place in neonatal care and that they approach difficulties in their work “potentially within a religious and spiritual rather than a uniquely secular framework” [27]. In such a stressful environment, Caldeira and Hall adds that “[s]piritual needs should be acknowledged within neonatal care whether these are of babies, families or the team itself” [28].

### Childbirth positive spiritual component

Our study reveals nonetheless a generally positive score for both parents. Affirmative answers to specific statements touching on the “appreciation of life’s beauty”, “respect”, “moral responsibility”, and “gratitude” reveal the strong positive spiritual potential of childbirth in someone’s life. Research by Saroglou et al. also showed that despite much research on spirituality showing that negative events and emotions can increase spirituality, positive experiences such as “wonder at childbirth” can do the same [29]. Callister’s extensive ethnographic studies with new mothers also indicated “childbirth as a spiritually transforming experience”, “as a time to grow closer to God”, and “to make religiosity more meaningful” [5]. Our results indicated that this positive potential can be even higher for some mothers, specifically those with a religious affiliation or born outside of Canada. These results point out the relevance of “spirituality” as an object of study in regions like Quebec where religious practice has largely diminished but where spiritual beliefs have diversified [21]. We were surprised by the percentage of parents praying in the delivery room, a high number in the Quebec context. This may indicate that even in a medicalized society, where the priority is on safety and pain management, birth remains a truly intense (just over 6 h for vaginal delivery) and multidimensional life event where spirituality can emerge as an “intensification of the human” experience, as Dumas describes it [30].

### Spiritual score variations between mothers and fathers

This study is also the first to measure fathers’ spirituality in the delivery room, thus allowing a comparison with the mother’s experience. Three themes largely account for why mothers’ global scores were higher than fathers’ scores: “letting go”, “self-accomplishment”, and “meaningfulness”. Our qualitative data indicate that this might be related to the role fathers perceive they must play, where their contribution is perceived as one of active support for the mother. As such, the father is not focused on his own experience: “Gratitude toward my partner. As for myself, I was just there to support and encourage her.” Similarly, a phenomenological study on the experience of fathers in the delivery room by Longworth and Kingdon indicated a “lack of perceived control” and the struggle of fathers to define their role as main results [31]. Awareness of the “fragility of life” during childbirth was also indicated significantly more often by fathers. These differences might indicate a need for clinical adjustments in terms of the consideration of fathers’ spiritual needs during and after childbirth.

### Reactions toward questions about the spirituality of childbirth

Some comments by parents, especially the fathers’ comments, emerged unexpectedly during this study. Question marks were left (14 fathers, 2 mothers), humor was used (10 fathers, 2 mothers), but also -although rarely- aggressive reactions toward spirituality (3 fathers). This is especially true for themes more strongly associated with religious components, which were also ranked the lowest. These reactions can be a reflection of how spirituality is perceived in the general population and could indicate a “discomfort” with being questioned about spirituality in the public sphere. Interviews with parents in a previous qualitative phase of our research program revealed that an individual’ own spirituality was kept secret not only in public (e.g., at work) but also between partners as it was “not well seen” or “irrational” [32]. Fear of proselytism and confusion between religion and spirituality might also explain such reactions. McSherry, in her study on a model for advancing spirituality and spiritual care within nursing and health care practice, noted that this “discomfort” is not only from the patient’s point of view but also the caregiver’s [33]. In this sense, results that might appear contradictory in the mixed methods approach [25], such as parents giving similar comments below a statement but giving opposite quantitative answers, would be a perfect illustration of this complex relationship that parents can have with spirituality.

### Study limitations

The response rate in this study was higher than expected with over half of the questionnaires completed by both parents, but some specific groups might have been more prone to do so than others. Nonetheless, the survey did attempt to be exhaustive by being conducted seven days a week in the sole hospital in the region. Moreover, when compared to Quebec’s provincial data, the characteristics of our sample were similar to the population in terms of maternal age, parity, prematurity, and low-weight babies [34]. A second limitation was the subjective nature of parents’ answers. Results could vary because of negative aspects being underreported (e.g., a gratitude bias) or overestimated (e.g., high expectations), but also because of the level of fatigue normal following any childbirth. The time for the data collection might have also played a role, since the effects of some variables (e.g., severe perineal tear, baby in neonatal unit) can be felt more vividly few hours after delivery. A third limitation is possible data contamination if parents consulted each other while completing the survey. Finally, as described previously and despite our rigorous methodology, the subjective nature of the notion of “spirituality” remains a challenge for any study, and maybe even more when quantified. Nonetheless, the use of a mixed-methods design not only improved the description with the comments section but also in showing the limits of the questionnaire itself. The study of spirituality in healthcare settings remains moving: comments left by parents could be used to adjust following research.

### Perspectives

Future research could measure the impact of spiritual preparation and/or intervention during pregnancy on the spiritual experience of childbirth, or the impact of such experiences on the mode of parenting later developed. In clinical settings, maternity staff should better understand the spiritual dimension, and tools should be developed to support them, such as this spiritual assessment question suggested by Callister and Khalaf: “Do you have any spiritual beliefs that will help us better care for you?” [5].

## Conclusion

While previous qualitative studies have shown the spiritual dimension of childbirth for mothers in specific medical or cultural groups, our mixed-methods prospective approach in a public university hospital highlights a high spirituality score among a non-selected population with the pioneering quantitative data on both mothers’ and fathers’ spirituality prevalence in the delivery room. Spirituality does not only emerge from unordinary situations but also from any childbirth as an “intensification of the human” experience. A significantly higher score for mothers compared with fathers (notably for the spiritual themes of “meaningfulness”, “self-accomplishment”, and “letting go”) and fathers’ higher score in terms of “fragility of life” indicate the need to consider the spirituality of both parents, notably in the training of care teams.
